# The Impact of Perceived Organizational Politics on Peer Voice Endorsement: A Dual Mediation Model

**DOI:** 10.3390/bs15070892

**Published:** 2025-06-30

**Authors:** Peiwen Qiu, Tingjing Chen, Liao Hu, Hao Zhou

**Affiliations:** Business School, Sichuan University, Chengdu 610065, China; qiupeiwen@stu.scu.edu.cn (P.Q.); cherrychen1998@126.com (T.C.); fudaliao555@163.com (L.H.)

**Keywords:** perceived organizational politics, psychological safety, affective commitment, peer voice endorsement, cognitive-affective personality system theory

## Abstract

Peer voice endorsement, an emerging focus in voice research, is recognized for its important role in enhancing work efficiency. This study aims to examine the impact of perceived organizational politics on peer voice endorsement. It also explores the underlying mechanisms by developing a model based on the cognitive-affective personality system theory. Study data were collected in three waves from 226 full-time employees in China. The hypotheses were examined using SPSS 25.0 and the PROCESS macro. Empirical results indicate that perceived organizational politics negatively affects both psychological safety and affective commitment. Psychological safety and affective commitment also play a facilitating role in peer voice endorsement. Moreover, these two factors serve as mediators in the relationship between perceived organizational politics and peer voice endorsement. By highlighting the pivotal role of perceived organizational politics, this study offers valuable insights into its implications for peer voice endorsement. It further underscores the significance of psychological safety and affective commitment, demonstrating their essential function in cultivating a work environment that encourages peer voice endorsement.

## 1. Introduction

Employees are well-versed in their own and their department’s work and are most aware of the irrationalities in need of improvement within processes and systems. If employees can actively voice their opinions, it will not only help the organization to optimize processes and avoid losses but also stimulate innovation and change (Davidson et al., 2017; Liang et al., 2019). As research on voice progresses, researchers have found that merely encouraging employees to voice is far from sufficient (Morrison, 2014). Despite the benefits of employee constructive voice, the value of voice itself cannot be fully realized without its endorsement (Isaakyan et al., 2021; Zhang & Chen, 2024). Furthermore, W. Liu et al. (2010) proposed two types of voice behavior: speaking out, which is directed toward peers, and speaking up, which is directed toward leaders. Existing research on voice endorsement primarily focuses on speaking up, highlighting scenarios where leaders endorse voice (including those from employees and colleagues), which we refer to as managerial endorsement (Burris, 2012; Weiss & Morrison, 2019). Given that peers often serve as the primary recipients of voice, they may actively exchange ideas, share information, and express opinions regarding necessary improvements (Brykman & Raver, 2023; Detert et al., 2013). Yet, the interaction of peers is an aspect often overlooked within the context where much of the voice happens (Bain et al., 2021). Thus, different from managerial endorsement, this study examines peer voice endorsement, corresponding to speaking out.

Drawing on the cognitive-affective personality system (CAPS) theory proposed by Mischel and Shoda (1995), the dynamic interaction between cognitive-affective units and situational factors influences and shapes human behavior. Peer voice endorsement occurs within the organizational context, where an individual’s perception of the organizational environment plays a pivotal role in determining how they interpret peers’ voice behavior and decide whether to endorse their voice. Organizational politics, recognized as common self-serving behaviors within the organizational environment (C. H. Chang et al., 2009; H. Chang & Pak, 2024), can influence employees’ behaviors in the workplace through their perception of organizational politics, also known as perceived organizational politics (Vigoda, 2002). In the Chinese context, the phenomenon of organizational politics is notably more prevalent (Su & Xie, 2023), which is closely related to the cultural emphasis on interpersonal relationships. Guanxi, as a prominent cultural characteristic, profoundly shapes interpersonal interactions and power dynamics within organizations (Buckley et al., 2006). Moreover, Chinese culture emphasizes collectivism, valuing team harmony and collaboration among members (Bai et al., 2016). Within such a cultural environment, employees often exhibit increased sensitivity to organizational politics, such as power struggles and perceived injustice in resource distribution. Therefore, perceived organizational politics may have a more pronounced effect on employees in China than in Western countries. In this context, exploring the link between perceived organizational politics and peer voice endorsement presents an insightful opportunity to better understand organizational behavior across diverse cultural settings. In line with the CAPS theory, perceived organizational politics, serving as crucial social and interpersonal contextual cues, exerts an impact on peer voice endorsement. Specifically, perceived organizational politics affects peer voice endorsement by shaping individuals’ internal cognitive processes and affective states. Therefore, exploring the effect of perceived organizational politics on peer voice endorsement through both cognitive and affective lenses is essential.

Voice endorsement, while beneficial, also entails risks (Y. Cheng et al., 2020; Detert & Burris, 2007). Employees may find it challenging to gauge the efficacy of voice endorsement when they are uncertain about their peers’ motivations. Moreover, in highly politicized organizational environments, employees often perceive a threat to their status due to peers’ voice behavior and may hold negative views about the intentions behind their peers’ voice (P. Liu et al., 2022). From a cognitive perspective, when employees have a strong perception of organizational politics, they may feel uneasy about endorsing their peers’ voice. This unease could arise from a belief that their peers are involved in conspiracies, prompting them to avoid the risks of peer voice endorsement. From an affective standpoint, perceived organizational politics tends to diminish employees’ attachment to the organization. In environments characterized by heightened political behaviors, employees are less inclined to undertake risks that could potentially advance the organization’s growth. Consequently, by integrating cognitive and affective perspectives, this study examines how psychological safety and affective commitment function as mediators between perceived organizational politics and peer voice endorsement.

We therefore pose the following three research questions: (1) Does the subjective cognitive factor of perceived organizational politics influence employees’ psychological safety and affective commitment to the organization? If so, through what mechanisms? (2) How do employees’ psychological safety and affective commitment influence peer voice endorsement? (3) Does perceived organizational politics affect peer voice endorsement? If so, is the effect direct or indirect, and what mediating mechanisms are involved?

To conclude, this research investigates how employees’ perceptions of organizational politics influence their endorsement of peers’ voice, while also uncovering the psychological mechanisms that drive this relationship. Grounded in the CAPS theory, this study expands the scope of voice endorsement beyond the conventional leader-employee framework to the domain of peer voice endorsement. This study introduces organizational politics as the situational factor to examine peer voice endorsement, thereby enriching research on perceived organizational politics. Moreover, this study enhances our understanding of employee behavior and workplace engagement by clarifying the specific mechanisms through which psychological safety and affective commitment mediate the link between perceived organizational politics and peer voice endorsement.

The following sections are organized as follows: Section 2 provides a comprehensive review of the relevant literature and presents the research hypotheses. The data and methodology, as well as variable measurement, are presented in Section 3. In Section 4, we detail the data analysis methods and report the empirical results. Section 5 interprets the findings, discusses the theoretical contributions and practical implications, identifies study limitations, and suggests directions for future research. Section 6 concludes the study.

## 2. Literature Review and Hypotheses

### 2.1. POP, Psychological Safety, and Affective Commitment

Perceived organizational politics (POP) refers to how employees subjectively evaluate the self-serving actions of their colleagues and supervisors within the workplace, often attributing these behaviors to specific individuals (Ferris et al., 2000, 2019). Organizational politics often involves power struggles, informal norms, coalitions, and the pursuit of personal or group interests (C. Li et al., 2020). Previous research has generally viewed organizational politics negatively, believing that it adversely affects both organizational outcomes and employees’ work behaviors. For instance, Landells and Albrecht (2017) elaborated on the organizational-level impacts through qualitative research, highlighting phenomena such as unclear organizational goals, internal conflicts, and the prevalence of factions. At the individual level, perceived organizational politics is linked to emotions such as exclusion, frustration, and stress among employees. Al-Abrrow (2022) conducted a study on employees in the health sector, finding a positive association between their perceptions of organizational politics and silence. Through a survey of full-time employees at two private service enterprises in China, Zhou and Sun (2024) discovered a negative association between perceived organizational politics and employees’ positive affect. Additionally, the focus of research on perceived organizational politics has shifted from macro-level discussions to examining individual behaviors (Hochwarter et al., 2020). According to the CAPS theory, internal cognitive and affective units can be triggered by external situational factors, ultimately influencing individuals’ behaviors (Mischel & Shoda, 1995). Given the focus of this study on exploring peer voice endorsement, we direct our attention toward psychological safety as the cognitive factor and affective commitment as the affective factor.

Psychological safety refers to the perception that individuals can fully express their authentic selves without concern for potential harm to their reputation, status, or professional growth (Kahn, 1990). It also refers to a workplace condition that provides employees with sufficient certainty and predictability, allowing them to feel secure and trusted within the organization (Tu et al., 2019). However, the existence of organizational politics often leads to a high level of uncertainty, which conflicts with psychological safety. De Clercq et al. (2018) found in a study of ten organizations from various industries in Pakistan that organizational politics has a positive impact on employees’ perceptions of unfairness. Moreover, insufficient organizational fairness is one of the key reasons for employees’ lack of psychological safety (Chinomona & Chinomona, 2013). Specifically, in environments with high perceived organizational politics, limited organizational resources often lead internal individuals or interest groups to adopt informal means to maintain existing benefits or seek additional resources, resulting in unfairness and uncertainty in the organizational atmosphere (S. Wang et al., 2025). In such unpredictable situations, employees experience a general sense of unease, which in turn weakens their psychological safety (C. Li et al., 2020). Furthermore, when employees lack trust in their peers, they are more likely to interpret their peers’ actions as self-serving, intensifying tension. The uncertainty in interpersonal relationships and the work environment further diminishes employees’ psychological safety.

Affective commitment, widely regarded as the core dimension for measuring organizational commitment (Demircioglu, 2023; Im et al., 2016), reflects employees’ organizational identity, emotional attachment, loyalty, and involvement (Meyer & Allen, 1991). Existing studies have proved that individual characteristics (e.g., demographic traits and personal dispositions), organizational structure (e.g., decision-making decentralization, policy, and procedure formalization), and work experiences (e.g., organizational rewards, fairness in procedures, and support from supervisors) significantly impact employees’ affective commitment (Meyer & Allen, 1991). In a study of two private service enterprises in China, Zhou and Sun (2024) found that one of the key factors leading to the reduction of positive emotions is perceived organizational politics. When employees perceive high levels of organizational politics, it can signal several underlying issues: (a) it may indicate that the organization frequently employs power and resources for personal gain, leading to diminished trust among employees, heightened scrutiny of one another, reduced interpersonal interaction, and decreased collegial cohesion; (b) the organization might encounter ambiguity and uncertainty, hindering employees’ ability to grasp its goals and the methods to accomplish them; (c) it may suggest that organizational members engage in competition for limited resources for their individual benefit, thereby compromising organizational fairness and diminishing emotional attachment and commitment among employees. Perceived organizational politics is associated with employee burnout (Vigoda-Gadot & Talmud, 2010) and turnover intentions (C. H. Chang et al., 2009), both of which can serve as factors that undermine the emotional bond between employees and the organization.

To sum up, perceived organizational politics represents a hindrance stressor (C. H. Chang et al., 2009), and is detrimental to maintaining healthy employee-organization relationships. A politicized environment may jeopardize employees’ psychological safety and have a negative impact on their emotional bond with the organization. Consequently, Hypothesis 1 and Hypothesis 2 are proposed.

**Hypothesis** **1:** *Perceived organizational politics has a negative impact on employees’ psychological safety.*

**Hypothesis** **2:** *Perceived organizational politics has a negative impact on employees’ affective commitment.*

### 2.2. Psychological Safety, Affective Commitment, and Peer Voice Endorsement

In the existing literature, there is limited analysis of peer voice endorsement within organizations, with more focus on managerial endorsement (Lam et al., 2019; Wu et al., 2021). Previous research has shown that leaders may not always welcome the voice of subordinates and may reject it or even penalize employees with low-performance evaluations (Morrison, 2014). Additionally, researchers have begun to explore the risk factors influencing managerial endorsement (Burris, 2012; Detert & Burris, 2007). The risks of voice endorsement include: (a) the intention of the voicer is not clear, which may be a kind of criticism, threat, or even provocation, and voice endorsement may be seen as giving in and showing weakness to some extent; (b) voice endorsement often implies a desire to challenge the status quo, which can be risky and lead to negative outcomes such as disruption of workflow, increased costs, and decreased efficiency. Similarly, these risks are also applicable when it comes to peer voice endorsement. Compared to leaders, there is a lack of formal power relationship coordination mechanisms among employees, making peer voice endorsement more susceptible to factors such as emotions, relationships, and trust. Therefore, how to promote peer voice endorsement by enhancing employees’ psychological safety and affective commitment becomes an issue worth exploring.

Psychological safety empowers employees to engage in behaviors that might entail interpersonal risks, such as participating in open communication, voicing opinions and concerns, and actively seeking feedback (Pearsall & Ellis, 2011). In recent years, Mehmood et al. (2021) conducted a study using teams from the manufacturing sector in Pakistan and found that psychological safety is positively related to team creativity. Harvey et al. (2019) researched the sales teams of a large financial services firm in Canada and demonstrated that psychological safety positively influences team learning. Sherf et al. (2021) found, through a meta-analysis, that the psychological safety-silence relationship is stronger than the psychological safety-voice relationship. The voice behavior within the outcome variable of psychological safety has been extensively discussed at both the individual and team levels. Psychological safety plays a crucial role in enabling employees to express constructive ideas, offer improvement suggestions, and expose potential issues within the organization (Morrison, 2014; Mowbray et al., 2024). Individuals with a heightened sense of psychological safety perceive their peers as supportive of expressing their authentic selves and sharing ideas. This fosters an atmosphere of mutual respect, care, and positive affective cognition among peers. Employees collaborate effectively to resolve constructive conflicts and feel confident in taking personal risks (Edmondson, 1999). Consequently, they exhibit greater confidence in the ideas and suggestions put forth by their peers, viewing their voice behavior as aimed at enhancing work and avoiding errors. Additionally, employees with a strong sense of psychological safety are less apprehensive about the negative repercussions of work-related mistakes (Edmondson & Lei, 2014). Peer voice endorsement often involves challenging the status quo, which can lead to uncertain outcomes. However, those who possess a strong sense of psychological safety are more inclined to focus on problem-solving and improving work processes, rather than prioritizing self-protection (Edmondson, 2004). Therefore, employees who possess a heightened sense of psychological safety are more prone to respond positively to peers’ voice. They are inclined to evaluate the content thoughtfully, viewing it as an opportunity to enhance their work instead of perceiving it as a threat or additional burden.

Affective commitment refers to employees’ emotional attachment to the organization (Meyer & Allen, 1991). In existing research, affective commitment is associated with positive work behaviors. In a study involving employees from various industries, Shao et al. (2022) discovered that affective commitment positively influences job performance. Kim and Beehr (2020) conducted research on full-time employees in the United States and discovered that affective commitment contributes to a decrease in the occurrence of withdrawal behaviors. Employees with high levels of commitment to their organization typically accept the organization’s goals and values and pursue providing quality service on behalf of the organization (Bai et al., 2023; Shao et al., 2022). Moreover, when the organization effectively meets the needs of employees, their affective commitment toward the organization strengthens, fostering a deeper sense of identity and loyalty (Meyer & Allen, 1991). Caliskan et al. (2024) identified affective commitment as a key driver of voice behavior, suggesting that it not only deepens employees’ emotional attachment to the organization but also has a reinforcing effect on their likelihood to engage in voice behavior. Specifically, employees with strong affective commitment are more willing to share information within the organization (Tucker & Turner, 2015), prompting them to identify potential problems and improve work processes. They are also more inclined to endorse constructive voice from peers, thereby better achieving organizational goals. Therefore, we propose the following hypotheses.

**Hypothesis** **3:** *Employees’ psychological safety has a positive impact on peer voice endorsement.*

**Hypothesis** **4:** *Employees’ affective commitment has a positive impact on peer voice endorsement.*

### 2.3. The Mediating Role of Psychological Safety and Affective Commitment

According to the CAPS theory, situational factors have the potential to activate an individual’s cognitive-affective units, and the interaction between a situation and a cognitive-affective unit will further affect employees’ behavioral response to the situation (Mischel & Shoda, 1995). Perceived organizational politics is the subjective perception of political behavior in the organization, which also reflects the situational factor of organizational politics (Ferris et al., 2000). Psychological safety is a subjective feeling, referring to employees’ sense of environmental certainty and predictability within the organization, which enables them to freely express their opinions, take risks, and actively demonstrate initiative and creativity (Tu et al., 2019; Zhan et al., 2025). However, when there is a strong political atmosphere within the organization, employees face increased uncertainty and situational ambiguity, which in turn weakens their trust and sense of safety in the organizational environment (Edmondson, 1999). In such a context, individuals’ cognitive-affective units may be activated into a defensive psychological state, leading employees to exhibit heightened vigilance and avoidance tendencies when responding to peers’ voice (Saei & Liu, 2024). Specifically, if employees lack psychological safety, they may respond to peer voice with suspicion. On the one hand, they might suspect that personal motives lie behind the voice (Zhao et al., 2024). On the other hand, they may fear that endorsing such voice could threaten their own interests or organizational status. As a result, employees may be unwilling to genuinely endorse peers’ voice and fail to see the voice as an opportunity for problem-solving or self-improvement, thus suppressing the occurrence of constructive interactions within the organization.

The organization’s political behavior, characterized by breaches of operational rules, fosters uncertainty and distrust among employees (Kacmar & Carlson, 1997). The interpersonal relationships among peers will be negatively influenced, resulting in a decline in fair and just treatment. A study by Brammer et al. (2007) on employees of a large retail banking services company in the United Kingdom revealed a positive relationship between procedural fairness and organizational commitment. When employees perceive strong procedural fairness in their organization, they become more involved in their work and develop stronger affective commitment (Stan & Vîrgă, 2021). Affective commitment is gradually formed through employees’ work experiences in interactions with leaders, work teams, and others (Meyer & Allen, 1997). Consequently, organizational politics, as a situational factor, triggers responses within employees’ affective units. The interaction between organizational politics and affective units impacts employees’ organizational behavior. Low levels of affective commitment diminish employees’ sense of identity and involvement, resulting in reduced motivation to pursue organizational objectives. Therefore, when facing the voice behavior of peers, employees will not easily endorse their voice for the sake of the organization. Moreover, supporting peers’ voice will deplete employees’ resources, both tangible (e.g., time and attention) and intangible (e.g., emotional energy and personal resources) (Zhang & Chen, 2024). When employees perceive political behavior within the organization, their affective commitment tends to decrease, as they feel that their position and contributions within the organization are overlooked or undervalued. In such situations, employees are typically reluctant to increase their work input to make greater contributions to achieving organizational goals (Malik et al., 2024). They may believe that even if they work hard, they will not receive the recognition and rewards they deserve.

**Figure 1:**
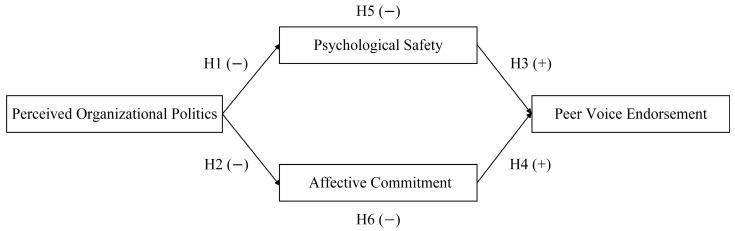
Theoretical model.

In summary, high levels of perceived organizational politics reflect a pervasive political climate within an organization. As shown in Figure 1, this heightened political atmosphere activates employees’ cognitive units regarding psychological safety and also impacts affective units, specifically their affective commitment to the organization. By influencing the cognitive-affective units, perceived organizational politics ultimately impacts employees’ evaluation of peer voice endorsement. Therefore, Hypothesis 5 and Hypothesis 6 are proposed.

**Hypothesis** **5:** *Psychological safety mediates the relationship between perceived organizational politics and peer voice endorsement.*

**Hypothesis** **6:** *Affective commitment mediates the relationship between perceived organizational politics and peer voice endorsement.*

## 3. Method

### 3.1. Sample and Procedure

The data were collected through the administration of a questionnaire survey. During the study period, participants were registered as on-the-job Master of Business Administration (MBA) students at a university in Western China. They were full-time employees from various industries and companies, with diverse professional backgrounds and work experiences. To mitigate common method bias, the study employed a questionnaire survey conducted offline three times with a two-week interval between each administration. At Time 1, the independent variable and control variables—including demographic variables and Zhongyong—were measured. The mediating variables—psychological safety and affective commitment—were measured at Time 2, while the dependent variable—peer voice endorsement—was evaluated at Time 3. Each time participants completed the questionnaire, they provided their cell phone number. These numbers served as identifiers to match the three questionnaires. Additionally, participants received a 10 CNY phone credit reward for each completed questionnaire. Those who completed all three questionnaires received an additional 10 CNY in phone credit as an incentive.

Initially, 409 questionnaires were distributed. Following data matching and the elimination of invalid responses, 226 valid questionnaires were collected, yielding a response rate of 55.3%. Table 1 presents the basic characteristics of the participants. Among the participants, 120 were male (53.1%) and 106 were female (46.9%). The average age of participants was 32.2 years, and their average organizational tenure was 6.02 years.

### 3.2. Measures

All measures were translated into Chinese following the procedure of translation and back-translation (Brislin, 1980). Participants responded using a 5-point Likert scale, except for the demographic variables.

Perceived organizational politics. The scale developed by Vigoda (2001) consists of six items. A sample item is “People in this organization attempt to build themselves up by tearing others down”. In this study, the Cronbach’s alpha was 0.89.

Psychological safety. This scale, which comprises seven items, was originally developed by Edmondson (1999). A sample item is “No one in this organization would deliberately act in a way that undermines my efforts”. The Cronbach’s alpha for this scale in the current study was 0.80.

Affective commitment. We assessed affective commitment using a five-item scale from Gao-Urhahn et al. (2016). A sample item is “I am glad to have joined this organization”. The Cronbach’s alpha for this scale was 0.94.

Peer voice endorsement. Peer voice endorsement was measured by a five-item scale adapted from Burris (2012). One of the sample items is “I will implement the comments and suggestions from my colleagues”. In this study, the Cronbach’s alpha of the scale was 0.85.

Control variables. This study controlled for employees’ gender, age, organizational tenure, and Zhongyong. Since the research sample is from China, cultural factors may influence the study results. The concept of Zhongyong, in the context of Chinese culture, is expressed through individuals’ tendency to adopt the principle of “harmony in diversity” when engaging in interpersonal relationships. This means adopting a neutral and balanced approach when facing differing opinions or interacting with people of diverse personalities (He & Li, 2021). Therefore, to mitigate the impact of Zhongyong on the research findings and enhance the generalizability, Zhongyong is also included as part of the control variables. The six-item scale developed by Du et al. (2014) was employed to measure Zhongyong, with a Cronbach’s alpha of 0.80.

## 4. Results

### 4.1. Confirmatory Factor Analysis and Common Method Bias Testing

Confirmatory factor analysis was employed in this study. As shown in Table 2, the hypothesized five-factor model exhibited a satisfactory fit with the data (*χ*^2^/*df* = 1.73, RMSEA = 0.06, CFI = 0.91, IFI = 0.91, TLI = 0.90), while alternative models demonstrated poor fit. This finding confirmed the distinctiveness of the five measures. Although we collected data at three different time points, the data all originated from self-reports by employees, which still posed a potential risk of common method bias. Therefore, Harman’s single-factor test was conducted, and the results showed that the first factor accounted for 28.76% of the total variance, below the threshold of 40% (Podsakoff et al., 2003). This suggests that common method bias is not a serious concern.

### 4.2. Descriptive Statistics and Correlation Analysis

Table 3 presents the mean values, standard deviations, and correlation coefficients for all variables. Data analysis results indicated a significant negative correlation between perceived organizational politics and psychological safety (*r* = −0.32, *p* < 0.01), as well as between perceived organizational politics and affective commitment (*r* = −0.56, *p* < 0.01). A significant positive correlation was observed between psychological safety and peer voice endorsement (*r* = 0.30, *p* < 0.01). Additionally, affective commitment was positively correlated with peer voice endorsement (*r* = 0.35, *p* < 0.01). These results serve as preliminary support for the testing of the following hypotheses.

### 4.3. Hypothesis Testing

In this study, SPSS version 25.0 was utilized to test the hypotheses. As shown in Table 4, perceived organizational politics exhibited a significant negative impact on psychological safety (*β* = −0.32, *p* < 0.001), thus supporting Hypothesis 1. Similarly, perceived organizational politics significantly negatively impacted affective commitment (*β* = −0.55, *p* < 0.001), confirming Hypothesis 2. Psychological safety demonstrated a significant positive effect on peer voice endorsement (*β* = 0.16, *p* < 0.05), which validated Hypothesis 3. Furthermore, affective commitment had a significant positive impact on peer voice endorsement (*β* = 0.30, *p* < 0.001), thereby supporting Hypothesis 4.

To further verify Hypothesis 5 and Hypothesis 6, this study employed the PROCESS macro proposed by Hayes (2013). As shown in Table 5, the mediating effect of psychological safety between perceived organizational politics and peer voice endorsement was found to be significant (indirect effect = −0.06, 95% CI = [−0.10, −0.03], excluding 0). Therefore, Hypothesis 5 was supported. Additionally, the mediating effect of affective commitment between perceived organizational politics and peer voice endorsement was significant (indirect effect = −0.13, 95% CI = [−0.19, −0.08], excluding 0). Consequently, Hypothesis 6 was supported. The data analysis presented above supported all the hypotheses proposed in this study, offering robust empirical evidence for the suggested relationships. A summary of the hypothesis testing results can be found in Table 6.

## 5. Discussion

This study uses CAPS theory to investigate whether and how perceived organizational politics influences peer voice endorsement. Unlike the existing studies, which mainly focused on voice endorsement between leader and subordinate, this study pays attention to voice endorsement between peers.

The results highlight a detrimental impact of perceived organizational politics on psychological safety, aligning with the findings of J. Li et al. (2014), who found the same negative relationship in Chinese electronic companies. However, this study extends voice behavior to peer voice endorsement, thereby providing additional insights and enriching the existing literature. We also found that perceived organizational politics negatively affects affective commitment. Previous studies have verified the negative relationship between the two (Kimura, 2013; Vigoda-Gadot & Talmud, 2010). This study further confirms this perspective. Moreover, we propose that psychological safety promotes peer voice endorsement. In previous studies, Sherf et al. (2021) suggested that psychological safety predicts silence but does not predict voice behavior. Xu et al. (2019) found that psychological safety is positively correlated with subordinates’ voice. Building on prior research, this study further extends the field by highlighting the positive effect of psychological safety on peer voice endorsement. Affective commitment has also been shown to exert a positive influence on peer voice endorsement, consistent with previous findings. For example, Tucker and Turner (2015) reported that employees who possess ideas on improving occupational safety and demonstrate strong affective commitment are more likely to proactively propose improvements and voice their opinions. J. Cheng et al. (2022) also demonstrated the positive relationship between affective commitment and employees’ voice behavior through a survey of employees from 15 retailing companies located along the southeastern coast of China. This research provides additional evidence supporting the positive impact of affective commitment on peer voice endorsement. The results reveal that perceived organizational politics inhibits peer voice endorsement by influencing psychological safety and affective commitment separately. According to J. Li et al. (2014), psychological safety functions as a key mediator connecting perceived organizational politics with voice behavior. C. Liu et al. (2025) proposed that the relationship between technical anonymity and employees’ willingness to speak up was partially mediated by psychological safety. Additionally, Landells and Albrecht (2019) suggested that further research could examine how psychological safety functions as either a mediator or moderator in the relationship between perceived organizational politics and a variety of outcome variables. Therefore, this study enriches previous scholars’ research and responds to their call. Moreover, affective commitment serves as a mediator in the relationship between leadership style and voice behavior (J. Cheng et al., 2022). Morrison (2023) also highlighted in the review that affective commitment is a key mediating variable in the relationship between leadership and voice behavior. Building on this, the present study extends the role of affective commitment by proposing that it not only serves as a mediating variable in the relationship between different leadership styles and voice behavior, but also acts as a crucial intermediary between perceived organizational politics and peer voice endorsement. The findings increase our understanding of the pathways through which perceived organizational politics impacts peer voice endorsement and provide new insights into how to promote peer voice endorsement.

### 5.1. Theoretical Contributions

First, this study extends the scope of voice endorsement research. Existing research on voice endorsement has primarily examined how leaders respond to employees’ voice, with particular emphasis on the role of leadership styles on managerial endorsement (Ng et al., 2021; Shu et al., 2022). As enterprises modernize and organizational structures flatten, they promote horizontal information flow among colleagues and enhance interpersonal communication (Manz, 1986). Also, the dispersion of knowledge and resources across the organization leads to a pivotal role in exchange relationships among increasingly interdependent colleagues (Argote, 2024; Burt, 2005). Furthermore, existing research on peer voice endorsement primarily focuses on the behavioral reactions of peers as bystanders after observing an employee’s voice being endorsed by the leader (Huai et al., 2024; Ni et al., 2024; Poulton et al., 2024). Building on the above, this study shifts focus from leader-subordinate to peer voice endorsement, providing empirical evidence to enhance peer voice endorsement in organizations.

Second, this study introduces perceived organizational politics as a cognitive contextual factor in the examination of peer voice endorsement, enriching the existing literature on perceived organizational politics. Earlier research has largely emphasized the adverse effects of perceived organizational politics on voice behavior (Bergeron & Thompson, 2020; C. Li et al., 2020). However, these studies often overlook the fact that voice behavior can be directed toward different targets, each with distinct motivational bases and behavioral implications (Zhou & Sun, 2025). Such an oversight may lead to a limited perspective. Given that the target of voice can influence both the intent and response to voice behavior, it is theoretically valuable to explore how perceived organizational politics affects employees’ willingness to endorse their peers’ voice. By incorporating peer voice endorsement into the study of perceived organizational politics, this research extends current work on perceived organizational politics and voice in a novel direction. Moreover, voice endorsement is a form of social persuasion where the persuader aims to influence the persuadee through compelling information (Liang et al., 2024; Whiting et al., 2012). Classic persuasion models highlight key factors, such as the persuader, the persuadee, the message, and the context (Y. Wang et al., 2021). Past research has focused on the characteristics of the voice sender, the message, and its delivery (Bonaccio & Dalal, 2006; Howell et al., 2015; Milliken et al., 2003), but has neglected the role of the organizational context, in particular the impact of political perceptions. By incorporating perceived organizational politics into the study, this research shows how a politically charged environment affects individuals’ willingness to endorse peers’ voice, enhancing our understanding of voice endorsement mechanisms in organizations.

Third, this study examines psychological safety and affective commitment as two mediating variables to explore the mechanisms through which perceived organizational politics influences peer voice endorsement, from both cognitive and affective perspectives. The findings provide preliminary evidence that psychological safety and affective commitment represent two potential pathways between perceived organizational politics and peer voice endorsement. While previous research on the effects of perceived organizational politics on employee behavioral outcomes has mainly drawn on theories such as social exchange theory (N. A. Khan et al., 2019) and conservation of resources theory (Zhou & Sun, 2025), this study adopts a different theoretical perspective by employing the CAPS theory to examine the mediating mechanisms. In line with CAPS theory, cognitive-affective units are activated by situational factors, and the interaction between these units shapes employees’ behavioral choices. When an organization is politically charged, employees are often exposed to uncertainty, ambiguous conditions, and a lack of clarity in rules and processes, which can make them feel insecure (C. H. Chang et al., 2009; Y. N. Cheng et al., 2024). In such environments, employees may adopt self-protective behaviors or form cliques due to power struggles and perceived injustice. Some might even retaliate against peers they see as contributing to the political climate (A. Khan & Chaudhary, 2022; Meisler et al., 2019), which weakens their affective commitment to the organization. As a result, organizational politics can reduce employees’ willingness to endorse constructive voice aimed at improving the organization. This study proposes a parallel mediation model, extending CAPS theory and offering new insights into the link between perceived organizational politics and peer voice endorsement.

### 5.2. Practical Implications

The present study provides valuable practical insights, in addition to theoretical contributions. The results suggest that employees’ psychological safety and affective commitment are weakened in a politically charged organizational environment, further hindering peer voice endorsement. Organizational politics, as perceived by employees, is a detrimental aspect of the work environment. It is often associated with negative job attitudes, diminished motivation, and increased work disengagement (Hochwarter et al., 2020). Therefore, to create a positive organizational atmosphere and enhance employees’ job satisfaction and motivation, organizations, leaders, and employees must work together and take effective measures to reduce the negative impact of political behaviors, thereby promoting the establishment of a more open and healthy communication and collaboration environment.

#### 5.2.1. Practical Implications for Organizations

Organizations must recognize the negative aspects of organizational politics, as ambiguity and high uncertainty in organizational contexts can create space conducive to political behaviors. Therefore, organizations should reduce such uncertainty by standardizing processes and improving rules and regulations, providing clear guidance for employees to follow in their work (H. Chang & Pak, 2024). Additionally, organizations should focus on enhancing employees’ psychological safety and affective commitment levels, as these are key factors influencing peer voice endorsement. To achieve this, organizations can foster a supportive culture centered on trust, open communication, and meaningful work. Moreover, they can enhance employee engagement through team-building activities to foster stronger connections between employees and the organization (A. Khan & Chaudhary, 2022). Through these measures, organizations can not only enhance employee engagement but also create a healthier and more positive work environment, ultimately improving organizational effectiveness and employees’ long-term development.

#### 5.2.2. Practical Implications for Leaders

For leaders, they should lead by example and establish appropriate behaviors through their role as a model. On the one hand, leaders should reduce ambiguity in the workplace by increasing communication with employees, ensuring that employees receive necessary information in a timely manner, and clearly communicating expected behavioral standards. In doing so, leaders can not only minimize the impact of organizational politics but also help employees align with organizational goals (Kaur & Kang, 2022). On the other hand, leaders can eliminate unreasonable or unwritten rules that might lead to unnecessary conflicts, punish harmful political activities, and reduce employees’ motivation to engage in organizational politics (C. Li et al., 2020). Additionally, leaders should provide necessary support for employees’ work, trust them, and reduce their sense of insecurity. Leaders should appropriately empower employees, enhance their sense of involvement and trust, and increase their affective commitment to the organization, thereby creating favorable conditions for peer voice endorsement.

#### 5.2.3. Practical Implications for Employees

Employees should recognize that experiencing organizational politics is inevitable (Malik et al., 2024). Therefore, they should adopt a positive attitude toward organizational politics and be prepared to respond effectively. Organizational politics is not always negative (Ferris et al., 2019; Zhou & Sun, 2024). It can also provide opportunities for growth and development. The key lies in how employees identify, interpret, and respond to these political behaviors. In an environment influenced by organizational politics, employees should cultivate the ability to effectively recognize, assess, and leverage opportunities, thereby enhancing their social sensitivity and interpersonal skills (McAllister et al., 2018). Additionally, employees should acknowledge the importance of peer voice endorsement. As the partners they interact with most frequently in daily work, peers have a clearer insight into an individual’s work status and progress, and are better positioned to identify problems and areas for improvement in the work process. By endorsing peers’ voice, employees can not only detect and resolve issues more quickly but also continuously improve their work methods, leading to enhanced work efficiency and performance.

### 5.3. Limitations and Directions for Further Research

First, the study is constrained by sample limitations. As the sample comprises only full-time employees from Western China, the applicability of the conclusions may be limited in other cultural contexts. Future research could consider expanding the sample to groups from different cultural backgrounds and conducting cross-cultural comparisons to verify the impact of cultural factors on the research findings. Second, the measurement of variables in this study relies on employee self-reporting. Despite efforts to minimize common method bias by collecting data at three distinct time points, the issue cannot be entirely ruled out. Moreover, the questionnaire survey used in this study cannot adequately explain the causal relationships between variables. Future studies could collect data from a variety of sources (e.g., supervisor evaluations or organizational records) to mitigate the bias and employ more research methods, such as experimental studies, to verify causality. Finally, this study may involve variables at the individual (e.g., personality traits) and group (e.g., group climate) levels that could moderate the relationships between the key variables. Therefore, the inclusion of these potential moderating variables should be considered in future research.

## 6. Conclusions

Overall, this study explores the relationship between perceived organizational politics and peer voice endorsement from the cognitive and affective perspectives, providing valuable insights into how organizational factors influence peer voice endorsement. The results indicate that perceived organizational politics significantly undermines employees’ psychological safety and affective commitment. Furthermore, both psychological safety and affective commitment are crucial factors in fostering peer voice endorsement. Further analysis reveals that psychological safety and affective commitment mediate the relationship between perceived organizational politics and peer voice endorsement, uncovering the influencing pathway that has not been sufficiently explored in previous research. Additionally, these findings emphasize the crucial role of both cognitive and affective factors in promoting peer voice endorsement. By recognizing and effectively addressing these factors, organizations and leaders can create an environment that mitigates the negative impact of organizational politics, thus encouraging employees to express their views and suggestions and endorse constructive voice. In summary, this study deepens our understanding of how organizational politics affects employees’ voice behaviors and provides valuable theoretical and practical guidance for organizations and leaders who aim to motivate employees to express opinions and endorse voice, while fostering a positive work environment.

## Figures and Tables

**Table 1:** The distribution of basic characteristics of participants.

Items	Category	Frequency	Percentage
Gender	Male	120	53.1%
	Female	106	46.9%
Age	Below 30	111	49.1%
	31−40	95	42.0%
	41−50	19	8.4%
	Above 50	1	0.5%
Organizational tenure	Below 1 year	32	14.2%
	1−3 years	48	21.2%
	3−5 years	51	22.5%
	5−10 years	63	27.9%
	Over 10 years	32	14.2%

Note. *N* = 226.

**Table 2:** Results of confirmatory factor analysis.

Model	Model Structure	*χ^2^*	*df*	*χ*^2^/*df*	RMSEA	CFI	IFI	TLI
Five-factor model	POP; PS; AC; PVE; ZY	786.32	454	1.73	0.06	0.91	0.91	0.90
Four-factor model 1	POP + ZY; PS; AC; PVE	1135.16	458	2.48	0.08	0.82	0.82	0.81
Four-factor model 2	POP; PS + AC; PVE; ZY	946.26	458	2.07	0.07	0.87	0.87	0.81
Three-factor model	POP + ZY; PS + AC; PVE	1293.18	461	2.81	0.09	0.78	0.78	0.76
Single factor model	POP + PS + AC + PVE + ZY	2256.83	464	4.86	0.13	0.53	0.53	0.49

Note. *N* = 226. POP = perceived organizational politics; PS = psychological safety; AC = affective commitment; PVE = peer voice endorsement; ZY = Zhongyong.

**Table 3:** Means, standard deviations, and correlations.

Variables	Mean	SD	1	2	3	4	5	6	7	8
1. Gender	1.47	0.50	-							
2. Age	32.20	5.24	−0.28 **	-						
3. Organizational tenure	6.02	4.87	−0.10	0.61 **	-					
4. Zhongyong	4.09	0.47	0.07	0.06	0.13	(0.80)				
5. POP	2.52	0.88	−0.05	−0.17 *	−0.02	−0.03	(0.89)			
6. Psychological safety	3.32	0.64	−0.11	0.11	0.10	0.20 **	−0.32 **	(0.80)		
7. Affective commitment	3.58	0.92	−0.05	0.22 **	0.09	0.16 *	−0.56 **	0.54 **	(0.94)	
8. Peer voice endorsement	3.65	0.56	0.07	0.12	0.04	0.20 **	−0.13	0.30 **	0.35 **	(0.85)

Note. Gender, 1 = male; 2 = female. * *p* < 0.05; ** *p* < 0.01.

**Table 4:** Results of mediated regression analyses.

Independent Variables	Model 1	Model 2	Model 3
	Psychological Safety	Affective Commitment	Peer Voice Endorsement
Gender	−0.15 *	−0.05	0.13 *
Age	−0.05	0.11	0.14
Organizational tenure	0.09	−0.01	−0.08
Zhongyong	0.20 **	0.14 **	0.11
POP	−0.32 ***	−0.55 ***	0.12
Psychological safety			0.16 *
Affective commitment			0.30 ***
*R* ^2^	0.16	0.36	0.18
*F*	8.56 ***	24.38 ***	6.88 ***

Note. *N* = 226. * *p* < 0.05; ** *p* < 0.01; *** *p* < 0.001.

**Table 5:** Results of indirect effect analyses.

Indirect Path	Indirect Effect	LLCI	ULCI
POP-Psychological Safety-Peer Voice Endorsement	−0.06 *	−0.10	−0.03
POP-Affective Commitment-Peer Voice Endorsement	−0.13 *	−0.19	−0.08

Note. *N* = 226. Bootstrap sample size = 5000. * *p* < 0.05.

**Table 6:** Summary of the hypotheses.

Hypothesis Number	Hypothesis Statement	Supported/Not Supported
H1	Perceived organizational politics has a negative impact on employees’ psychological safety.	Supported
H2	Perceived organizational politics has a negative impact on employees’ affective commitment.	Supported
H3	Employees’ psychological safety has a positive impact on peer voice endorsement.	Supported
H4	Employees’ affective commitment has a positive impact on peer voice endorsement.	Supported
H5	Psychological safety mediates the relationship between perceived organizational politics and peer voice endorsement.	Supported
H6	Affective commitment mediates the relationship between perceived organizational politics and peer voice endorsement.	Supported

## Data Availability

The data from this study are available from the corresponding author upon reasonable request.
